# Dollar-Yuan Battle in the World Trade Network

**DOI:** 10.3390/e25020373

**Published:** 2023-02-17

**Authors:** Célestin Coquidé, José Lages, Dima L. Shepelyansky

**Affiliations:** 1 Équipe Physique Théorique et Astrophysique, Institut UTINAM, Université Bourgogne Franche-Comté, CNRS, 25000 Besançon, France; 2 Laboratoire de Physique Théorique, Université de Toulouse, CNRS, UPS, 31062 Toulouse, France

**Keywords:** international trade, commercial flows, Markov chains, complex networks, opinion formation model

## Abstract

From the Bretton Woods agreement in 1944 till the present day, the US dollar has been the dominant currency in world trade. However, the rise of the Chinese economy has recently led to the emergence of trade transactions in Chinese yuan. Here, we mathematically analyze how the structure of international trade flows would favor a country to trade whether in US dollar or in Chinese yuan. The trade currency preference of a country is modeled as a binary variable with the properties of a spin in an Ising model. The computation of this trade currency preference is based on the world trade network built from the 2010–2020 UN Comtrade data and is determined by two multiplicative factors: the relative weight of trade volume exchanged by the country with its direct trade partners and the relative weight of its trade partners in global international trade. The performed analysis, based on the convergence of the Ising spin interactions, shows that from 2010 to present a transition took place, and the majority of the world countries would now have a preference to trade in Chinese yuan if one only considers the world trade network structure.

## 1. Introduction

In 1944, the Bretton Woods agreements established a system of payments based on the US dollar (USD). The USD effectively became the world currency, i.e., the standard to which every other currency was pegged [1]. Till now, the USD remained the dominant world trade currency, and, as an example, the United Nations (UN) reports world trade transactions between countries in USD [2]. However, the possible end of the dollar dominance is increasingly discussed as the Chinese yuan (CNY) is gradually becoming credible as a reserve currency [3,4,5,6,7,8,9]. Moreover, recently important trade transactions were considered to be realized in CNY instead of USD, such as oil sales from Saudi Arabia to China [10]. Thus, the growth of the Chinese economy [11] opens up the possibility for a country to prefer the CNY to the USD for their trade exchanges.

The World Trade Organization (WTO) Statistical Review [12] demonstrates the vital importance of international trade for the development and progress of the world’s countries. As the world economy deeply depends on world trade [13], the detailed analysis of the trade flows with a possible switch of the trade currency leads to significant effects on the world economy, which can be used by policy-makers and other stakeholders. Although the choice of the invoicing currency is generally taken at the importing/exporting firm’s level, for the sake of simplicity, our study is based on a model where each country, as a whole, takes the decision to trade in a given currency.

In the present paper, we model the trade currency preference of a country, i.e., here, whether a country prefers to trade in USD or in CNY, as a binary variable with the properties of a spin in an Ising model. In the absence of any political considerations or extra economical factors, here we consider that a country will prefer to trade in one currency rather than in the other one if the structure of the global international trade indeed favors the former. We study how the preference for a trade currency spreads from country to country with the help of a model derived from opinion formation models [14,15,16,17]. This currency preference model is applied to the world trade network (WTN) built from the 2010-to-2020 UN Comtrade data [2]. The constructed WTN describes trade relations between countries in terms of entropy flows. Our analysis, based purely on trade relations without taking account of external political factors or other considerations, clearly shows that, during this decade, a USD-to-CNY transition took place, which implies that the structure of the WTN would now favor trades in CNY rather than in USD.

## 2. Data Sets and Model Description

The network analysis of various types of flows finds many applications in various areas of science (see, e.g., [18,19,20]). More specifically, the network analysis of world trade transactions has been performed, e.g., in [18,21,22,23,24,25,26,27,28,29,30].

The WTN is constructed from the UN Comtrade database [2], which provides the money matrix M encoding the transactions of all the commodities between all the countries. The money matrix element $M_{c^{\prime }c}$ gives the total amount of commodities, expressed in USD of a given year, exported from country *c* to country $c^{\prime }$. The UN Comtrade database concerns 194 countries for the period 2010–2020. The Markov chain of trade transactions is characterized by two WTN trade matrices, S and $S^{*}$, whose elements are $S_{c^{\prime }c}=M_{c^{\prime }c}/M_{c}^{*}$ and $S_{c^{\prime }c}^{*}=M_{cc^{\prime }}/M_{c}$. Here, $M_{c}^{*}=\sum_{c^{\prime }}M_{c^{\prime }c}$ ($M_{c}=\sum_{c^{\prime }}M_{cc^{\prime }}$) gives the total amount of commodities exported from (imported to) country *c* to (from) the rest of the world. Consequently, the matrix element $S_{c^{\prime }c}$ ($S_{c^{\prime }c}^{*}$) gives the relative weight of the imports to (exports from) country $c^{\prime }$ from (to) country *c*. These matrix elements can be considered link weights of a directed network (see, e.g., [18]). By construction, the sum of each column of the matrices S and $S^{*}$ is equal to 1, i.e., $\sum_{c^{\prime }}S_{c^{\prime }c}=1$ and $\sum_{c^{\prime }}S_{c^{\prime }c}^{*}=1$, ensuring the fact that the stochastic processes of the trade transactions, both in export and import directions, belong to the class of Markov chains. Let us define, for a given year, the total world trade volume by $M=\sum_{c}M_{c}=\sum_{c}M_{c}^{*}$. Then, a given country, *c*, can be characterized by its import trade probability $P_{c}=M_{c}/M$ and its export trade probability $P_{c}^{*}=M_{c}^{*}/M$. These probabilities characterize the global capability of a given country to import and export, respectively. As an example, in 2019, the top 5 countries according to the import trade probability, $P_{c}$, are: 1. USA, 2. China, 3. Germany, 4. Japan, 5. UK and the top 5 Countries according to the export trade probability $P_{c}^{*}$ are: 1. China, 2. USA, 3. Germany, 4. Japan, 5. France.

In order to determine the trade currency preference (TCP) of country *c*, i.e., whether country *c* would prefer to trade in USD or in CNY with other countries, we assign to country *c* an Ising spin $\sigma_{c}=\pm 1$. The value $\sigma_{c}=-1$ (+1) indicates that country *c* prefers to trade in USD (CNY). Thus, we obtain a network of interacting Ising spins, each one attached to a country. We define the interaction energy of country (spin) *c* as

(1) Ec=12∑c′≠cσc′Sc′c*+Sc′c*Pc′*+Pc′*

which is the sum of all the energies of interaction of country (spin) *c* with its direct trade partners (spins) $c^{\prime }$. Country *c* TCP possibly flips from one currency to the other according to the sign of the computed $E_{c}$: If $E_{c}<0$, then country *c* spin becomes $\sigma_{c}=-1$ (preference to trade in USD) and if $E_{c}>0$, then country *c* spin becomes $\sigma_{c}=+1$ (preference to trade in CNY). From the interaction energy $E_{c}$ expression (1), the formation of country *c* TCP is sensitive to the TCPs of the direct commercial partners through the term $\sigma_{c^{\prime }}$, weighted by two important factors:

- The “Sc′c*+Sc′c*” term, which encodes the relative strength of the import-export flows between country *c* and a direct trade partner, $c^{\prime }$. As an example, in 2010, 0.4% of the total volume of exports from Russia was imported by Brazil ($S_{\mathrm{BR},\mathrm{RU}}=0.004$), and 1.9% of the total volume of exports from Brazil was imported by Russia ($S_{\mathrm{RU},\mathrm{BR}}=0.019$). Also, 1.7% of the total volume of imports to Russia was exported from Brazil ($S_{\mathrm{BR},\mathrm{RU}}^{*}=0.017$) and 1.0% of the total volume of imports to Brazil was exported from Russia ($S_{\mathrm{RU},\mathrm{BR}}^{*}=0.010$).
- The “Pc′*+Pc′*” term, which encodes the global trade capability of partner $c^{\prime }$ in the WTN. As an example, in 2010, the total imports to Brazil and to Russia represented 1.3% ($P_{\mathrm{BR}}=0.013$) and 1.7% ($P_{\mathrm{RU}}=0.017$) of the total world trade volume *M*, and the total exports from Brazil and from Russia represented 1.5% ($P_{\mathrm{BR}}^{*}=0.015$) and 3.1% ($P_{\mathrm{RU}}^{*}=0.031$) of the total trade volume *M*.

Consequently, as an example, the direct contribution of China to the possible change in TCP of Russia comes from the (SCN,RU*+SCN,RU*)(PCN*+PCN*) term in (1), which changes from 0.01 in 2010 to 0.014 in 2019, indicating a significant increase in relative trade values between Russia and China. This 40% increase is partly the reason why, in 2010, Russia’s TCP still depended on the initial distribution of the TCPs over all countries (in 2010, Russia belonged to the hereafter defined swing group, see the Section 3) and in 2019, Russia’s TCP is always CNY independently on the initial TCPs distribution (in 2019, Russia belongs to the hereafter defined CNY group, see the Section 3). Of course, Russia’s TCP also depends on the other (Sc′c*+Sc′c*)(Pc′*+Pc′*) terms in (1) associated with countries other than China, $c^{\prime }$.

**Figure A1:**
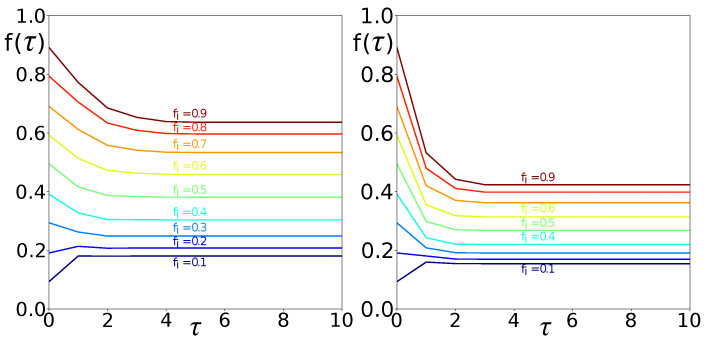
Evolution of the averaged fraction f(τ) of countries with USD trade preference as a function of the number of Monte Carlo shake steps τ for the years 2010 (**left** panel) and 2019 (**right** panel). The average is performed over $N_{r}=10^{4}$ spin configurations. The colors indicate the initial fraction $f_{i}$ of countries with USD trade preference at τ=0.

In this model, we keep USA and China always trading in USD and CNY, respectively. We start with an initial fraction $f_{i}$ of randomly chosen countries that prefer to trade in USD; we assign a −1 value to their spins. Consequently, the complementary fraction $1-f_{i}$ of countries prefer to trade in CNY, we assign a +1 value to their spins. A first Monte Carlo shake allows us to determine the energy $E_{c_{1}}$ for a randomly picked spin $\sigma_{c_{1}}$. The spin $\sigma_{c_{1}}$ flips or not according to the sign of the newly computed $E_{c_{1}}$, and, consequently, the TCP of country $c_{1}$ either stays the same or possibly changes from one currency to the other (i.e., from USD to CNY or from CNY to USD). Then, with the obtained new configuration of spins, a second shake is performed for another randomly chosen spin $\sigma_{c_{2}}$$\left(c_{2}\neq c_{1}\right)$, and so on. After 192 shakes, the trade currency preference for each country is determined (USA and China spins are always kept fixed). The ensemble of these shakes forms the first time step τ=1. We observe that after at most five consecutive time steps (τ>5), the system converges to a fixed steady-state configuration of spins, which stays unchanged for higher values of τ. This procedure is applied to $N_{r}=10^{4}$ initial random spin configurations with a fixed fraction $f_{i}$ of countries with an initial TCP for USD. We follow the evolution with τ of the fraction f(τ) of countries preferring to trade in USD till the steady-state $f_{f}$ at τ=10 is obtained. The complementary fraction $1-f_{f}$ gives then the fraction of countries preferring to trade in CNY once the steadystate is reached. An example of time evolution f(τ) is shown in Figure A1 of Appendix A.

The above-described TCP formation model applied on the WTN is similar to models of opinion formation on social networks used to study voting systems, strike phenomena, coalition formation (see, e.g., [14,15] for reviews), or opinion propagation in the WWW or Twitter [16]. Our model belongs to the class of Ferromagnetic Ising spin [31] models previously used to describe opinion dynamics [14,15,16,17] on regular and complex networks. Usually, an Ising spin, with a randomly chosen value −1 or +1, is assigned to each node. The possible flip of a spin, i.e., the possible change in the opinion of a node of the network, taken at random, possibly affects the opinion of its direct neighbors. As in Ferromagnetic Ising spins models at low temperatures, after multiple avalanches of spin flips, a long-range order is established all over the network. As a consequence, one or more giant homogeneous opinion clusters are formed, inside which the nodes behave collectively. Possibly, by percolation, one of the opinion clusters dominates and spans most of the network. A review can be found in, e.g., Chapter 8 of [15].

In the frame of Ferromagnetic Ising spins models, it is very natural that a spin oriented up and surrounded by spins oriented down will also be flipped down [14,15,16,17]. This physical process is directly implemented in the opinion formation models [14,15,16,17], and we also use this flip rule in our mathematical model based on Equation (1). Indeed, if a country has mainly trade partners preferring to trade in CNY, it naturally would prefer to also trade in CNY instead of USD. Such an approach to the analysis of opinion formation in social networks is broadly used in the literature [14,15,16,17], and we simply extend and apply it here to the WTN.

## 3. Results

**Figure 1:**
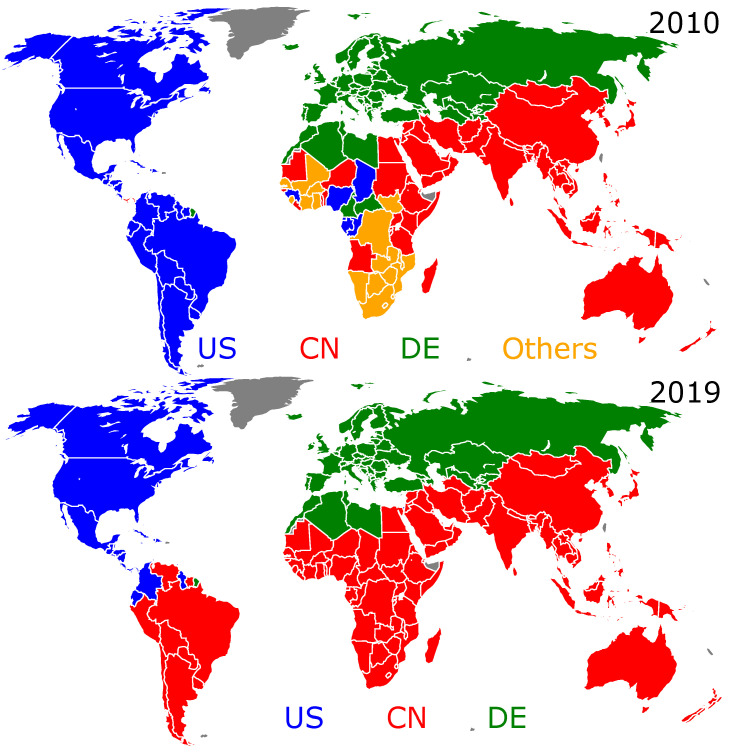
Geographical distribution of clusters in the WTN for the years 2010 (**top** panel) and 2019 (**bottom** panel) was obtained using the Louvain modularity method [32] with Dugué’s algorithm [33]. Each cluster is labeled using the ISO2 code of the country with the best import and export trade probabilities $P_{c}$ and $P_{c}^{*}$. The leaders of the clusters are the USA (blue), China (red), and Germany (green). The cluster Others (gold) gathers other small-size clusters.

**Figure A2:**
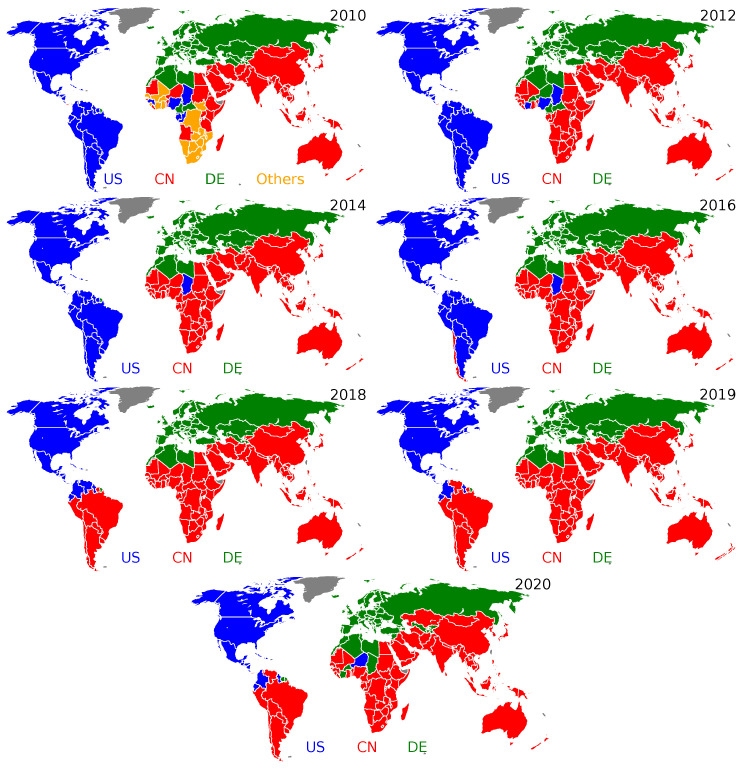
Geographical distribution of clusters in the WTN for the years 2010, 2012, 2014, 2016, 2018, 2019, and 2020 obtained using the Louvain modularity method [32] with Dugué’s algorithm [33]. Each cluster is labeled using the ISO2 code of the country with the best import and export trade probabilities $P_{c}$ and $P_{c}^{*}$. For all the considered years, the leaders of the clusters are the USA (blue), China (red), and Germany (green). The cluster Others (gold) gathers other small-size clusters.

As a preliminary step, let us characterize, for the years 2010 and 2019, the WTN using the Louvain method for cluster detection [32,33]. The results presented in Figure 1 show the existence of three main clusters formed around the USA, China, and Germany. We observe that from 2010 to 2019, the size of the cluster around China extends significantly. Indeed, from 2010 to 2019, the US-cluster loses almost all of the South American countries to the benefit of the CN-cluster, and a dominant part of Africa enters the CN-cluster. Meanwhile, the cluster formed around Germany remains practically unchanged, including the EU countries, the countries of the former Soviet Union, and most of the Maghreb. Examples of WTN clustering for other years of the considered decade are shown in Figure A2 of Appendix A.

**Figure 2:**
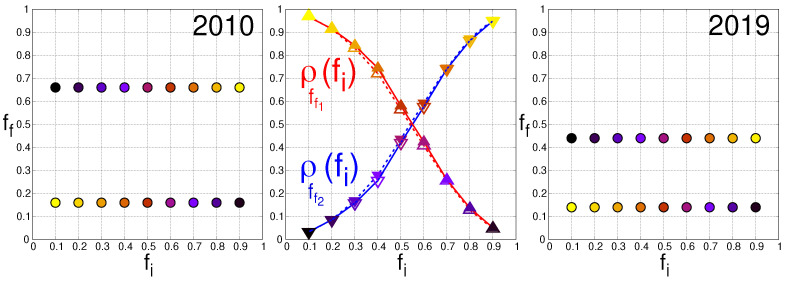
Final fraction $f_{f}$ of countries preferring to trade in USD as a function of the initial fraction $f_{i}$ for the years 2010 (**left** panel) and 2019 (**right** panel). Left and right panels: for any initial fraction $f_{i}$, the Monte Carlo procedure converges toward one of two final fractions $f_{f}$. These two final fractions are $f_{f_{1}}=0.16$ and $f_{f_{2}}=0.66$ in 2010 (**left** panel) and $f_{f_{1}}=0.14$ and $f_{f_{2}}=0.44$ in 2019 (**right** panel). The color of a point ($f_{i}$,$f_{f}$) indicates the portion $\rho_{f_{f}}\left(f_{i}\right)$ of the $N_{r}=10^{4}$ initial configurations, with a corresponding initial fraction $f_{i}$, which attains the final state with the corresponding final fraction $f_{f}$. The color ranges from black for $\rho_{f_{f}}\left(f_{i}\right)=0$ (all the countries preferring to trade in CNY rather than in USD) to bright yellow for $\rho_{f_{f}}\left(f_{i}\right)=1$ (all the countries preferring to trade in USD rather than in CNY). Middle panel: Portion $\rho_{f_{f}}\left(f_{i}\right)$ of the $N_{r}=10^{4}$ initial configurations, the fraction $f_{i}$ of which initially prefers to trade in USD, which attains the final state with the final fraction $f_{f}$. The red (blue) curve and the up (down) triangles correspond to the lowest (highest) value $f_{f_{1}}$ ($f_{f_{2}}$) of the two final fractions $f_{f}$. The full (empty) symbols correspond to the year 2019 (2010).

However, this preliminary cluster analysis of the WTN, based on the maximization of the modularity [32,33], does not determine the trade preference of the countries for either USD or CNY. The Monte Carlo procedure, described in the previous section, allows us to obtain the final fraction $f_{f}$ of world countries that prefer to trade in USD and, conversely, the final fraction $1-f_{f}$ of world countries that prefer to trade in CNY. Figure 2 shows the final fraction $f_{f}$ as a function of the fraction $f_{i}$ of countries that initially prefer to trade in USD. For each value of $f_{i}$, we randomly picked $N_{r}=10^{4}$ different configurations of spins. We observe that for any initial fraction $f_{i}$ belonging to the interval [0,1], only two final fractions $f_{f_{1}}$ and $f_{f_{2}}$ can be reached. However, the probability that a given spin configuration reaches one or the other final fraction values depends on the initial distribution of the TCPs over the countries. Let us take $f_{f_{1}}<f_{f_{2}}$. Quite naturally, the higher (lower) the initial fraction $f_{i}$ is, the higher the probability to obtain the highest (lowest) final value $f_{f_{2}}$ ($f_{f_{1}}$) is. The middle panel of Figure 2 gives, for the years 2010 and 2019, the probabilities $\rho_{f_{f_{1}}}\left(f_{i}\right)$ and $\rho_{f_{f_{2}}}\left(f_{i}\right)$ of obtaining the final fraction $f_{f_{1}}$ and $f_{f_{2}}$ as a function of the initial fraction $f_{i}$. In 2010, see the left panel of Figure 2, each of the final fractions corresponded to a majority of countries with either a USD preference ($f_{f_{2}}=0.66$) or a CNY preference ($f_{f_{1}}=0.16$). This is no longer the case in 2019, see the right panel of Figure 2, for which the two final fractions $f_{f_{1}}=0.14$ and $f_{f_{2}}=0.44$ are below 0.5 and give both a CNY preference for the majority of the world countries. In one decade, and according to the sole structure of the WTN, we pass from a bipolar USD-CNY trade currency preference to a global domination of the CNY.

**Figure 3:**
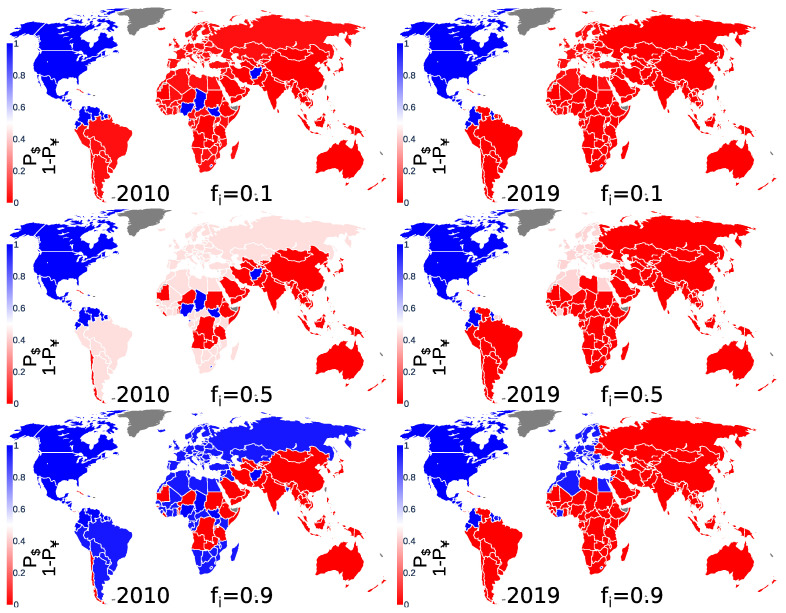
World distribution of the trade currency preference probability for the years 2010 and 2019. Each panel corresponds to a given year and a given fraction $f_{i}$ of countries preferring to initially trade in USD. Left (right) column panels correspond to the year 2010 (2019). The top, middle, and bottom rows correspond to $f_{i}=0.1$, 0.5, and 0.9, respectively. Each country is characterized by a probability $P_{\$}$ to obtain a USD trade preference at the end of the Monte Carlo procedure. The CNY trade preference probability of a country is then $P_{￥}=1-P_{\$}$. The color ranges from red for $P_{\$}=0$ and $P_{￥}=1$ to blue for $P_{\$}=1$ and $P_{￥}=0$. The average TCP probability $P_{\$}$ has been computed from $N_{r}=10^{4}$ random initial TCP distributions.

Let us define the TCP probability $P_{\$}\left(c\right)$ to obtain, for country *c*, a USD preference at the end of the Monte Carlo procedure. The probability of obtaining, for country *c*, a CNY preference is then $P_{(}c)=1-P_{\$}\left(c\right)$. The probability $P_{\$}$ is obtained from the application of the Monte Carlo procedure to the $N_{r}=10^{4}$ initial TCP distributions. Figure 3 shows the TCP probability world distribution. Figure 3 left panels illustrate the above-described bipolar USD-CNY trade currency preference that existed in 2010: for high (low) $f_{i}$, most of the countries finally prefer USD (CNY). In 2019, shown in Figure 3 right panels, the CNY dominance is clearly observed. Indeed, even for high $f_{i}$, most of the countries finally prefer CNY over USD.

**Figure 4:**
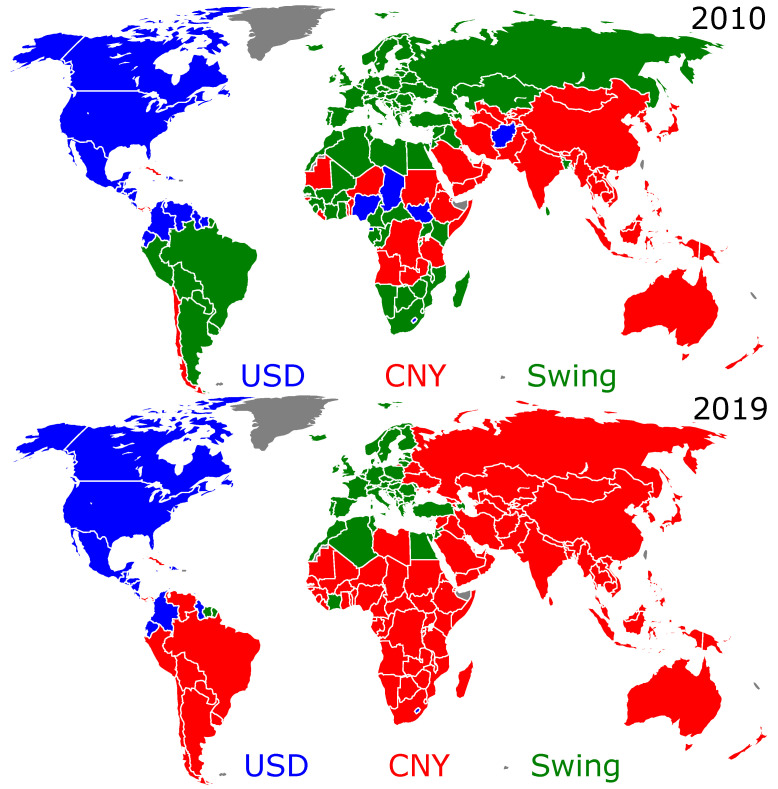
World distribution of countries belonging to the USD group (blue, hard preference to trade in USD), the CNY group (red, hard preference to trade in CNY), and the swing group (green, the TCP can change between USD and CNY depending on the initial conditions). The world maps are shown for the years 2010 (**left** panel) and 2019 (**right** panel).

**Figure A3:**
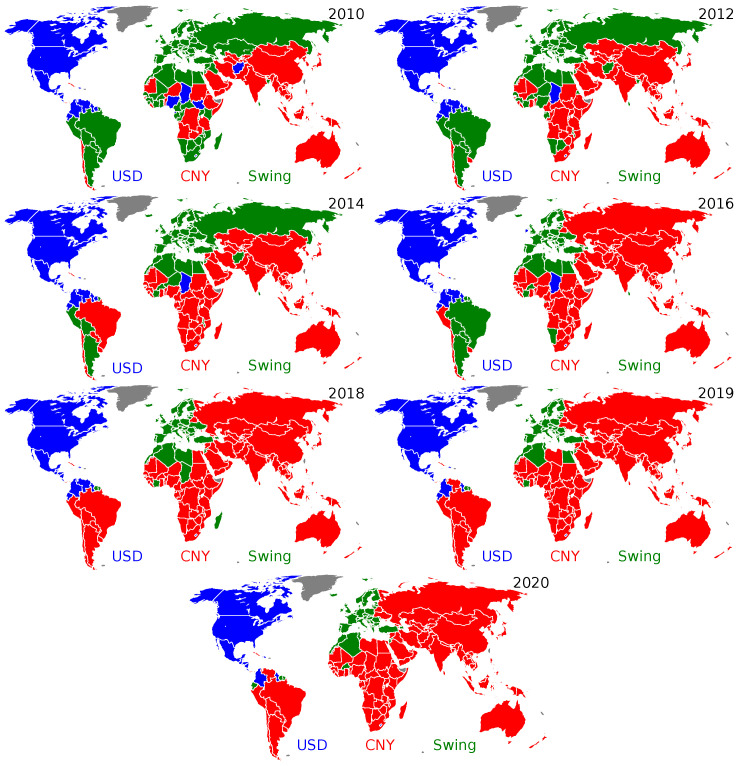
World map distribution of countries belonging to the USD group (blue, hard preference to trade in USD), the CNY group (red, hard preference to trade in CNY), and the swing group (green, the TCP can change between USD and CNY depending on the initial conditions). The world maps are shown for the years 2010, 2012, 2014, 2016, 2018, 2019, and 2020.

The reason for the bistability of the final outcomes $f_{f}$ (see $f_{1}$ and $f_{2}$ in Figure 2) can be understood from the analysis of the distribution of the USD or CNY trade preference over the countries. In fact, there are two groups of countries that keep, for any initial fraction $f_{i}$, a hard preference to trade in USD (the USD group) and in CNY (the CNY group). Otherwise stated, a country of the CNY (USD) group, independently of its initial TCP and of the initial TCPs of the other countries, will always end up in the CNY (USD) group. A third group of swing states (the swing group) may change their TCP. Amazingly, depending on the initial configuration of countries that prefer to trade in USD or in CNY, the countries belonging to this swing group collectively adopt, at the equilibrium, either the USD or the CNY as trade currency. These swing states are collectively responsible for the final outcome: if they adopt USD (CNY) at the equilibrium, the final fraction $f_{f}$ will be the highest (lowest) of the two possible fractions, i.e., $f_{2}$ ($f_{1}$). These three groups are shown on the world maps displayed in Figure 4 for the years 2010 and 2019 (the world maps for the other years of the past decade are shown in Figure A3 of Appendix A). We clearly see that there is a drastic change from 2010 to 2019: a large number of countries passed from the swing group to the CNY group, which has considerably increased in size during the last decade. Indeed, the former Soviet Union countries, almost all of South America and Africa belong, in 2019, to the CNY group. In contrast, from 2010 to 2019, the size of the USD group reduced only slightly, loosing a few countries: Venezuela, Nigeria, Chad, South Sudan, Equatorial Guinea, Afghanistan, and the Federated States of Micronesia switched to the CNY group and Suriname and Israel to the swing group. In 2019, the swing group is mainly composed of EU countries, the UK and some Mediterranean countries (Turkey, Egypt, Morocco, Algeria, Tunisia, and Israel). The lists of the countries belonging to the USD, CNY, and swing groups in 2010 and in 2019 are given in Table A1, Table A2, Table A3, Table A4, Table A5 and Table A6 of Appendix A.

**Figure 5:**
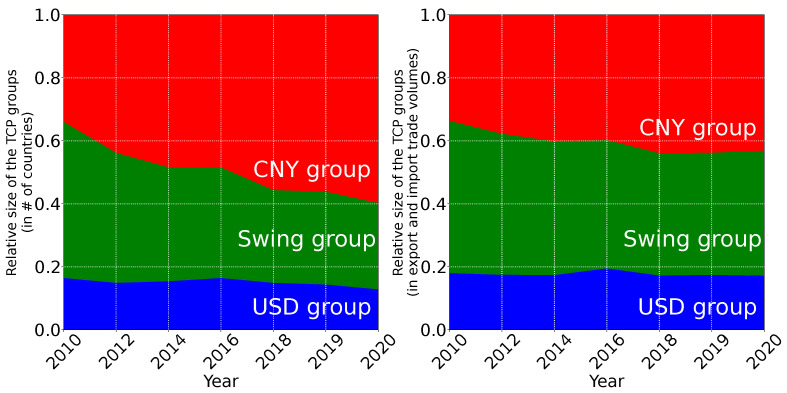
Time evolution of the sizes of the trade currency preference groups. The three bands correspond to the USD group (blue), the CNY group (red), and the swing group (green). The width of a band corresponds to the size of the corresponding group expressed as: (**left** panel) the ratio between the countries belonging to the group and the total number of countries, (**right** panel) the ratio between the total trade volume exchanged by the countries of the group and the total volume exchanged by all the world countries. The trade volumes are expressed in USD for the concerned year. The # symbol stands for “number”.

The time evolution over the last decade of the size of the three TCP groups is shown in Figure 5. The left panel of Figure 5 displays the fraction of countries belonging to each group, and the right panel displays the fraction of the total volume of import and export exchanged by each group. From Figure 5, left panel, we observe that the CNY group (red band) steadily grows along the decade from a fraction of 34% of the countries in 2010 to 60% in 2020. This growth of the CNY group is mainly compensated by the depletion of the swing group (green band), whose size drops from 50% of the countries in 2010 to 27% in 2020. Meanwhile, the size of the USD group (blue band) slightly decreased from 16% of the countries in 2010 to 13% in 2020. The trends are the same but less pronounced for the fraction of the trade volume exchanged by the different groups (see Figure 5, right panel). The fraction of the trade volume exchanged by the CNY (swing) group increases (decreases) from 34% (49%) in 2010 to 43% (40%) in 2020. Meanwhile, the fraction of the trade volume exchanged by the USD group stayed quite constant during the decade (17% for both 2010 and 2020). Consequently, the number of countries switching during the last decade from the swing group to the CNY group represents 23% of countries but represents only 9% of world trade volume. The CNY club increased but with somewhat less important new entrants in terms of trade volume exchanged.

**Figure A4:**
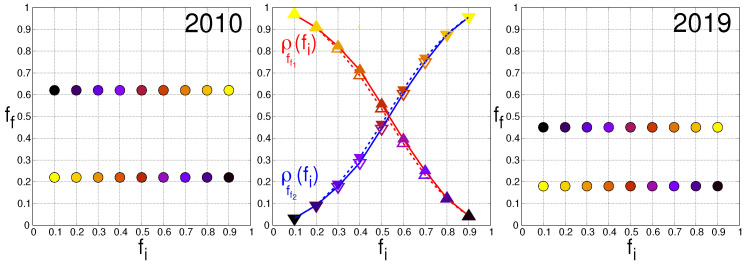
Same as Figure 2 but always keeping USA, Canada, UK, Australia, and New Zealand trading in USD and China, Brazil, Russia, India, and South Africa in CNY. The final fraction $f_{f}$ of countries preferring to trade in USD as a function of the initial fraction $f_{i}$ for the years 2010 (**left** panel) and 2019 (**right** panel). Left and right panels: for any initial fraction, $f_{i}$, the Monte Carlo procedure converges toward one of two final fractions $f_{f}$. These two final fractions are $f_{f_{1}}=0.22$ and $f_{f_{2}}=0.62$ in 2010 (**left** panel) and $f_{f_{1}}=0.18$ and $f_{f_{2}}=0.45$ in 2019 (**right** panel). The color of a point ($f_{i}$,$f_{f}$) indicates the portion $\rho_{f_{f}}\left(f_{i}\right)$ of the $N_{r}=10^{4}$ initial configurations, with a corresponding initial fraction $f_{i}$, which attains the final state with the corresponding final fraction $f_{f}$. The color ranges from black for $\rho_{f_{f}}\left(f_{i}\right)=0$ (all the countries preferring to trade in CNY rather than in USD) to bright yellow for $\rho_{f_{f}}\left(f_{i}\right)=1$ (all the countries preferring to trade in USD rather than in CNY). Middle panel: Portion $\rho_{f_{f}}\left(f_{i}\right)$ of the $N_{r}=10^{4}$ initial configurations, the fraction $f_{i}$ of which initially prefers to trade in USD, which attains the final state with the final fraction $f_{f}$. The red (blue) curve and the up (down) triangles correspond to the lowest (highest) value of the two final fractions $f_{f}$. The full (empty) symbols correspond to the year 2019 (2010).

**Figure A5:**
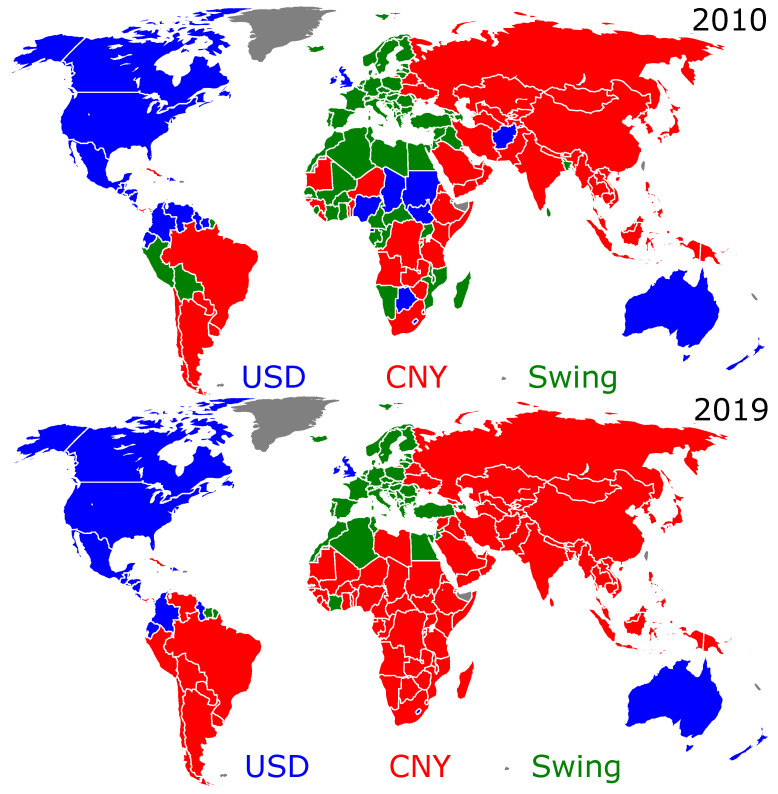
Same as Figure 4 but always keeping USA, Canada, UK, Australia, and New Zealand trading in USD and China, Brazil, Russia, India, and South Africa in CNY. World map distribution of countries belonging to the USD group (blue, hard preference to trade in USD), the CNY group (red, hard preference to trade in CNY), and the swing group (green, the TCP can change between USD and CNY depending on the initial conditions). The world maps are shown for the years 2010 (**top** panel) and 2019 (**bottom** panel).

**Figure A6:**
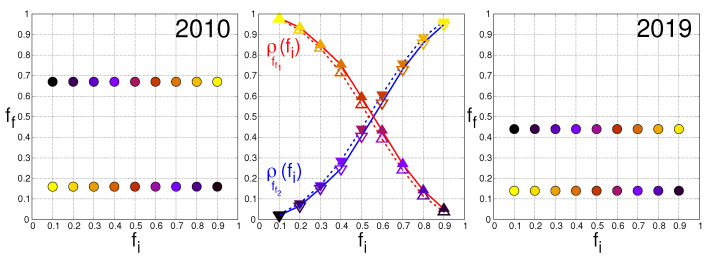
Same as Figure 2 but replacing the import trade probabilities $P_{c}$ and the export trade probabilities $P_{c}^{*}$ in (1) by the PageRank and the CheiRank probabilities obtained from the Google matrix of WTN. Final fraction $f_{f}$ of countries preferring to trade in USD as a function of the initial fraction $f_{i}$ for the years 2010 (**left** panel) and 2019 (**right** panel). Left and right panels: for any initial fraction $f_{i}$, the Monte Carlo procedure converges toward one of two final fractions $f_{f}$. These two final fractions are $f_{f_{1}}=0.16$ and $f_{f_{2}}=0.67$ in 2010 (**left** panel) and $f_{f_{1}}=0.14$ and $f_{f_{2}}=0.44$ in 2019 (**right** panel). The color of a point ($f_{i}$,$f_{f}$) indicates the portion $\rho_{f_{f}}\left(f_{i}\right)$ of the $N_{r}=10^{4}$ initial configurations, with a corresponding initial fraction $f_{i}$, which attains the final state with the corresponding final fraction $f_{f}$. The color ranges from black for $\rho_{f_{f}}\left(f_{i}\right)=0$ (all the countries preferring to trade in CNY rather than in USD) to bright yellow for $\rho_{f_{f}}\left(f_{i}\right)=1$ (all the countries preferring to trade in USD rather than in CNY). Middle panel: Portion $\rho_{f_{f}}\left(f_{i}\right)$ of the $N_{r}=10^{4}$ initial configurations, the fraction $f_{i}$ of which initially prefers to trade in USD, which attains the final state with the final fraction $f_{f}$. The red (blue) curve and the up (down) triangles correspond to the lowest (highest) value of the two final fractions $f_{f}$. The full (empty) symbols correspond to the year 2019 (2010).

**Figure A7:**
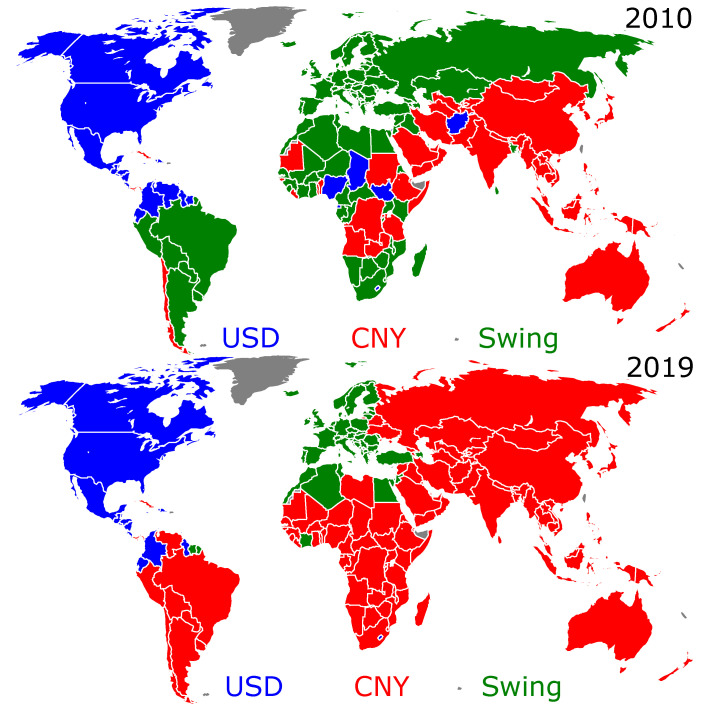
Same as Figure 4 but replacing the import trade probabilities $P_{c}$ and the export trade probabilities $P_{c}^{*}$ in (1) by the PageRank and the CheiRank probabilities obtained from the Google matrix of WTN. World map distribution of countries belonging to the USD group (blue, hard preference to trade in USD), the CNY group (red, hard preference to trade in CNY), and the swing group (green, the TCP can change between USD and CNY depending on the initial conditions). The world maps are shown for the years 2010 (**top** panel) and 2019 (**bottom** panel).

Let us note that we obtain practically the same results if, instead of keeping China always trading in CNY and the USA always trading in USD, we keep China and the other BRICS (Brazil, Russia, India, and South Africa) always trading in CNY and the USA and other Anglo-Saxon countries (Canada, UK, Australia, and New Zealand) always trading in USD; see, e.g., Figure A4 and Figure A5 in Appendix A, which are quite similar to Figure 2 and Figure 4. This result asserts the dominance of China and the USA in the world trade network. We have also considered replacing the import trade probabilities $P_{c}$ and the export trade probabilities $P_{c}^{*}$ in (1) with the PageRank and the CheiRank probabilities obtained from the Google matrix of the WTN. These probabilities, which allow the measuring of the capability of a country to import or export products throughout the WTN, were used to analyze international trade [28,29,30]. Such a replacement of the probabilities, i.e., of the centrality measures of the WTN, leads again to practically the same results (see, e.g., Figure A6 and Figure A7 in Appendix A, which are similar to Figure 2 and Figure 4).

## 4. Conclusions and Discussion

The question addressed in the current work is the following one: assuming that only the WTN structure matters, what would be the trade currency preference for each country? As a first step, we used a model with two currencies associated with the nowadays leading economies, i.e., USA and China. A next step toward a more refined model would be to consider additional currencies, such as the EUR for the eurozone. The world economy being strongly polarized, it is illusory to consider more than three dominant trade currencies. The world partition obtained by a naive application of the Louvain modularity method on the bare WTN supports this assertion (see Figure 1). Whether or not the swing group, which in 2019 is reduced to the eurozone and other EUR-dependent economies (see Figure 4), crystallizes into a stable EUR group is an open question. The possible stability of a three trade-currency model is another question. We leave these questions for subsequent work. The results presented here for the CNY-USD trade currency model should capture the main features of more refined models.

In conclusion, our analysis, performed by superimposing an Ising spin network on the WTN, clearly shows that the structure of international trade would nowadays favor the main part of the world to trade in CNY, while in 2010, it would have favored trade in USD. We observe two final equilibrium states. In 2010, one of them characterized a USD preference and the other one a CNY preference, whilst in 2019, both of the two final states characterized a CNY preference. Nowadays, according to the WTN structure, for any initial distributions of countries preferring to trade in USD and in CNY, the final state would always favor a world that preferentially trades in CNY. The bistability of the final state is due to a group of swing states, which, depending on the initial distribution of the trade currency preferences over the countries, adopt, collectively, a preference for either USD or CNY. Of course, our analysis is based on the mathematical treatment of the trade flows between the world countries and does not take into account any geopolitical relations between the countries. However, it is often claimed that economics determines politics, and thus, we argue that the obtained results demonstrate drastic changes in the international trade structure, which now favors the yuan over the dollar.

These results, obtained from the intertwined structure of international trade flows, echo the current questioning about a hypothetical replacement of USD by CNY as the global currency [3,4,6,7,8,34] and the current trends consisting to label contracts in CNY for Saudi Arabia to China or Russia to China crude oil and petrol imports [8,9,10,35,36]. Although the road to the internationalization of the CNY is still long [37] and although some serious criteria, such as the transparency of China’s financial markets and the perceived-from-abroad-stability of the Chinese monetary policies, are still lacking to turn CNY into a global currency, our results nonetheless show that the international trade network is ready to harbor the USD vs. CNY competition and the possible USD to CNY transition. Hence, as the nowaday global trade flow structure favors CNY, interested economic stakeholders could be encouraged to bypass USD in order to establish contracts in CNY for a large variety of economic sectors going de facto beyond the current niche use of CNY in the crude oil and gas market.

## Data Availability

The data presented in this study are available on reasonable request from the corresponding author.

## Abbreviations

The following abbreviations are used in this manuscript:

CNY	Chinese yuan
TCP	Trade currency preference
UN	United Nations
USD	United States dollar
WTN	World trade network

## Appendix A

**Table A1:** List of the 32 countries belonging to the USD group in 2010. The trade currency preference of these countries is always USD at the end of the relaxation process. The countries are sorted by descending value of $\max\left(P_{c},P_{c}^{*}\right)$, i.e., the maximum value between the relative import volume $P_{c}$ and the relative export volume $P_{c}^{*}$, and, in the case of a tie, by descending value of $P_{c}^{*}$. The red (green) colored countries switched to the CNY (swing) group in 2019. The countries are represented by their ISO2 codes.

Countries of the USD Group in 2010							
1.	US	9.	GT	17.	JM	25.	GQ
2.	MX	10.	DO	18.	GY	26.	TD
3.	CA	11.	HN	19.	BS	27.	SS
4.	IL	12.	SV	20.	HT	28.	LC
5.	NG	13.	NI	21.	SR	29.	KN
6.	CO	14.	VE	22.	BB	30.	DM
7.	EC	15.	TT	23.	LS	31.	GD
8.	CR	16.	AF	24.	BZ	32.	FM

**Table A2:** List of the 66 countries belonging to the CNY group in 2010. The trade currency preference of these countries is always CNY at the end of the relaxation process. The countries are sorted by descending value of $\max\left(P_{c},P_{c}^{*}\right)$, i.e., the maximum value between the relative import volume $P_{c}$ and the relative export volume $P_{c}^{*}$, and, in the case of a tie, by descending value of $P_{c}^{*}$. The blue colored countries switched to the USD group in 2019. The countries are represented by their ISO2 codes.

Countries of the CNY Group in 2010							
1.	CN	18.	PK	35.	MR	52.	MV
2.	JP	19.	OM	36.	KG	53.	BT
3.	KR	20.	KH	37.	PG	54.	GM
4.	SG	21.	MM	38.	BJ	55.	DJ
5.	IN	22.	UZ	39.	CU	56.	SB
6.	VN	23.	BH	40.	YE	57.	TL
7.	AE	24.	CD	41.	LR	58.	ER
8.	MY	25.	AO	42.	RW	59.	GW
9.	TH	26.	ZM	43.	TM	60.	VU
10.	AU	27.	PA	44.	MH	61.	WS
11.	SA	28.	LA	45.	NP	62.	KI
12.	ID	29.	TZ	46.	TJ	63.	PW
13.	PH	30.	SD	47.	NE	64.	TV
14.	CL	31.	MN	48.	SO	65.	NR
15.	NZ	32.	BN	49.	KP	66.	TO
16.	KW	33.	ET	50.	CW		
17.	IR	34.	TG	51.	AG		

**Table A3:** List of the 96 countries belonging to the swing group in 2010. The trade currency preference of these countries is for all of them, either CNY or USD, at the end of the relaxation process. The countries are sorted by descending value of $\max\left(P_{c},P_{c}^{*}\right)$, i.e., the maximum value between the relative import volume $P_{c}$ and the relative export volume $P_{c}^{*}$, and, in the case of a tie, by descending value of $P_{c}^{*}$. The red (blue) colored countries switched to the CNY (USD) group in 2019. The countries are represented by their ISO2 codes.

Countries of the Swing Group in 2010							
1.	DE	25.	UA	49.	LY	73.	BF
2.	FR	26.	AR	50.	JO	74.	MG
3.	NL	27.	IQ	51.	PY	75.	GN
4.	IT	28.	BD	52.	CI	76.	ML
5.	UK	29.	KZ	53.	UY	77.	UG
6.	BE	30.	SI	54.	BA	78.	CG
7.	CH	31.	GR	55.	MZ	79.	MW
8.	ES	32.	PE	56.	MK	80.	PS
9.	RU	33.	EG	57.	KE	81.	FJ
10.	PL	34.	BG	58.	BO	82.	GA
11.	BR	35.	DZ	59.	NA	83.	SZ
12.	CZ	36.	QA	60.	IS	84.	SC
13.	TR	37.	MA	61.	MT	85.	SY
14.	AT	38.	LT	62.	CY	86.	ME
15.	SE	39.	BY	63.	SN	87.	SL
16.	HU	40.	RS	64.	BW	88.	BI
17.	ZA	41.	HR	65.	GE	89.	VC
18.	IE	42.	LU	66.	LB	90.	AD
19.	DK	43.	EE	67.	AL	91.	CV
20.	NO	44.	TN	68.	AM	92.	CF
21.	SK	45.	LV	69.	MD	93.	KM
22.	RO	46.	GH	70.	CM	94.	SM
23.	FI	47.	AZ	71.	MU	95.	ST
24.	PT	48.	LK	72.	ZW	96.	PN

**Table A4:** List of the 28 countries belonging to the USD group in 2019. The trade currency preference of these countries is always USD at the end of the relaxation process. The countries are sorted by descending value of $\max\left(P_{c},P_{c}^{*}\right)$, i.e., the maximum value between the relative import volume $P_{c}$ and the relative export volume $P_{c}^{*}$, and, in the case of a tie, by descending value of $P_{c}^{*}$. The red (green) colored countries belonged to the CNY (swing) group in 2010. The countries are represented by their ISO2 codes.

Countries of the USD Group in 2019							
1.	US	8.	DO	15.	BS	22.	LC
2.	MX	9.	HN	16.	HT	23.	VC
3.	CA	10.	SV	17.	BB	24.	KN
4.	CO	11.	NI	18.	LS	25.	WS
5.	EC	12.	TT	19.	BZ	26.	DM
6.	CR	13.	JM	20.	CW	27.	GD
7.	GT	14.	GY	21.	AG	28.	TO

**Table A5:** List of the 109 countries belonging to the CNY group in 2019. The trade currency preference of these countries is always CNY at the end of the relaxation process. The countries are sorted by descending value of $\max\left(P_{c},P_{c}^{*}\right)$, i.e., the maximum value between the relative import volume $P_{c}$ and the relative export volume $P_{c}^{*}$, and, in the case of a tie, by descending value of $P_{c}^{*}$. The blue (green) colored countries belonged to the USD (swing) group in 2010. The countries are represented by their ISO2 codes.

Countries of the CNY Group in 2019							
1.	CN	29.	IR	57.	BN	85.	SY
2.	JP	30.	PK	58.	CM	86.	NE
3.	KR	31.	OM	59.	VE	87.	SO
4.	SG	32.	KH	60.	ZW	88.	SL
5.	IN	33.	MM	61.	ET	89.	KP
6.	VN	34.	GH	62.	BF	90.	GQ
7.	RU	35.	UZ	63.	TG	91.	MV
8.	AE	36.	LK	64.	MR	92.	TD
9.	MY	37.	LY	65.	AF	93.	BI
10.	TH	38.	BH	66.	KG	94.	BT
11.	AU	39.	PY	67.	PG	95.	SS
12.	BR	40.	UY	68.	MG	96.	GM
13.	SA	41.	MZ	69.	BJ	97.	DJ
14.	ID	42.	KE	70.	GN	98.	SB
15.	ZA	43.	CD	71.	ML	99.	TL
16.	PH	44.	BO	72.	CU	100.	ER
17.	CL	45.	AO	73.	YE	101.	CF
18.	NG	46.	ZM	74.	UG	102.	GW
19.	UA	47.	NA	75.	LR	103.	KM
20.	AR	48.	PA	76.	RW	104.	VU
21.	IQ	49.	LA	77.	CG	105.	FM
22.	BD	50.	SN	78.	TM	106.	KI
23.	KZ	51.	TZ	79.	MW	107.	PW
24.	NZ	52.	BW	80.	MH	108.	TV
25.	PE	53.	GE	81.	NP	109.	NR
26.	KW	54.	SD	82.	GA		
27.	QA	55.	MN	83.	TJ		
28.	BY	56.	AM	84.	SZ		

**Table A6:** List of the 57 countries belonging to the swing group in 2019. The trade currency preference of these countries is for all of them, either CNY or USD, at the end of the relaxation process. The countries are sorted by descending value of $\max\left(P_{c},P_{c}^{*}\right)$, i.e., the maximum value between the relative import volume $P_{c}$ and the relative export volume $P_{c}^{*}$, and, in the case of a tie, by descending value of $P_{c}^{*}$. The blue colored countries belonged to the USD group in 2010. The countries are represented by their ISO2 codes.

Countries of the Swing Group in 2019							
1.	DE	16.	DK	31.	HR	46.	MD
2.	FR	17.	NO	32.	LU	47.	MU
3.	NL	18.	SK	33.	EE	48.	PS
4.	IT	19.	RO	34.	TN	49.	FJ
5.	UK	20.	FI	35.	LV	50.	SR
6.	BE	21.	PT	36.	AZ	51.	SC
7.	CH	22.	IL	37.	JO	52.	ME
8.	ES	23.	SI	38.	CI	53.	AD
9.	PL	24.	GR	39.	BA	54.	CV
10.	CZ	25.	EG	40.	MK	55.	SM
11.	TR	26.	BG	41.	IS	56.	ST
12.	AT	27.	DZ	42.	MT	57.	PN
13.	SE	28.	MA	43.	CY		
14.	HU	29.	LT	44.	LB		
15.	IE	30.	RS	45.	AL		
